# Development of a new craving index for anticipating quitting smoking in patients who undergo the Japanese smoking cessation therapy

**DOI:** 10.18332/tid/114164

**Published:** 2019-12-03

**Authors:** Chie Taniguchi, Hideo Tanaka, Sumie Nakamura, Sachiyo Saito, Hideo Saka

**Affiliations:** 1 College of Nursing, Aichi Medical University, Nagakute, Japan; 2 Clinical Research Center (CRC), Nagoya Medical Center (NMC), Nagoya, Japan; 3 Fujiidera Public Health Center, Fujiidera, Japan; 4 Department of Nursing, Nagoya Medical Center, Nagoya, Japan; 5 Department of Respiratory Medicine, Nagoya Medical Center, Nagoya, Japan

**Keywords:** craving, TCI, QSU-brief, quit smoking

## Abstract

**INTRODUCTION:**

The 10-item version of the Questionnaire of Smoking Urges (QSU-brief) has demonstrated excellent reliability. However, the QSU-brief may be too long to use in clinical settings. We developed a new craving index called the Tobacco Craving Index (TCI) and investigated how closely the TCI grade is associated with success of quitting smoking in Japanese smoking cessation therapy (SCT) patients.

**METHODS:**

The TCI questionnaire consists of two items: the first question asks about the strength of tobacco craving on a 4-point scale, and the second question asks about the frequency of tobacco craving per day on a 4-point scale. We conducted a prospective cohort study of 85 participants who underwent the Japanese SCT at a Japanese smoking cessation clinic. We administered the QSU-brief and TCI at each of the 5 sessions during the 12-week SCT.

**RESULTS:**

Significant correlations were observed between the TCI grade and QSU-brief score (r=0.27, 0.55, 0.72, 0.58 and 0.68, at the five sessions). The change in mean TCI grade showed a similar trend as the change in mean QSU-brief score among the 43 patients who succeeded in quitting smoking and also among the 7 patients who failed to quit smoking by the last session. Both TCI and QSU-brief assessed after the second session were significantly associated with the smoking status at the last session. The area under the receiver operating characteristic curve for the success of quitting smoking in TCI grade was 0.615–0.881 at the 5 sessions, whereas it was 0.536–0.849 in QSU-brief score.

**CONCLUSIONS:**

The TCI can be used as a predictive tool for success of quitting smoking in the Japanese SCT. As the TCI consists of two questionnaire items, it can be easily administered in smoking cessation interventions.

## INTRODUCTION

Craving, or strong desire, to use tobacco is one of the diagnostic criteria of Tobacco Use Disorder in DSM-V^1^ . Craving is defined by the irrepressible desire for substance consumption. Also, craving is often considered as a subjective motivational state in which an individual experiences an intense desire to engage in substance consumption^2^. Some studies claimed that craving is an independent predictor of smoking cessation, lapse, and relapse^3-5^. Hughes et al.^5^ conducted an internet survey to monitor the prevalence of craving among long-time abstinent smokers. They found that those who had prolonged craving showed lower quitting rate than those who had not prolonged craving^5^. Also, Treloar Padovano et al.^4^ examined factors associated with smoking lapses among adolescents and found that craving was an independent factor associated with lapse, relating to 15% increased odds of lapse^4^.

Japan has high tobacco consumption with over 20 million smokers^6^. At present, there are 17000 smoking cessation clinics covered by medical insurance in Japan. Patients receive smoking cessation treatment by a physician and counseling by nurses. Nurses’ counseling consists of reinforcement of motivation and self-efficacy for smoking cessation and discussing with patients how to cope with craving. Reese et al.^7^ investigated the association between abstinence self-efficacy and craving. They found that craving predicted lower abstinence self-efficacy in situations through increased craving motives. Therefore, it is important for physicians and nurses engaged in smoking cessation therapy (SCT) to pay attention to patients’ craving level.

The Japanese-translated version of the 10-item Questionnaire on Smoking Urges-Brief (QSU-brief) has been available in Japan^8,9^. In the QSU-brief, the respondent answers each item on a 7-point scale from strongly disagree to strongly agree^10^. Toll et al.^11^ performed a clinical trial of participants who underwent smoking cessation therapy by medication and suggested that greater decreases in craving as measured by the QSU-brief predicted low risk of smoking relapse. Although excellent reliability has been demonstrated^12^, the QSU-brief may be too long to use in clinical settings.

West et al.^13^ performed a laboratory study in which smokers were assigned to continue smoking or abstain completely for 24 hours, and compared various craving scales between the two groups. Their results suggested that the QSU-brief is not more sensitive to 24 hour forced abstinence or reliable than the Mood and Physical Symptoms Scale (MPSS) or a simple rating of craving (5-grade evaluation). However, their study did not assess the predictive power of craving scales for abstinence in a prospective design. There has been no study that compared craving scales in their ability to predict success of quitting smoking among patients who undergo smoking cessation therapy. In order to assess the degree of craving to use tobacco in smokers in clinical settings in Japan, it is important to develop an easy-to-use and reliable indicator of craving. Thus, we previously created a new index of tobacco craving called the ‘Tobacco Craving Index (TCI)’ that consists of the strength and frequency of craving in two questionnaire items^14^. Our previous study performed at six smoking cessation clinics using the TCI found that strong craving as assessed at the end of the SCT was associated with significantly increased risk of discontinuing cessation during the 12 months after the SCT (RR=0.85, 95% CI: 0.76–0.96)^14^. The aim of the present study was to investigate how closely the TCI grade is associated with success of quitting smoking in Japanese SCT patients and to compare the intensity of association for quitting smoking between the QSU-brief score and the TCI score.

## METHODS

### Study design and setting

We conducted a prospective cohort study to monitor the relationships between the TCI grade or QSU-brief score and smoking status at a Japanese smoking cessation clinic in Nagoya city. We administered two questionnaires including the TCI and QSU brief to evaluate craving of cigarette smoking. Participants filled in the questionnaires before each SCT session on a tablet-type device. The Japanese SCT covered by health insurance consists of a total of 5 sessions over a 12-week period; patients visit the clinic at 2, 4, 8 and 12 weeks after the initial visit. There were 2 physicians and 3 nurses at the clinic. At each session, the patient received smoking cessation treatment consisting of medication therapy from a physician (about 15 minutes). Varenicline, which is a partial agonist of the α_4_β_2_ subtype of nicotinic acetylcholine receptor, was the only medication used by the participants. After the physician’s treatment, a nurse gave specific advice including behavioral therapy for 20–30 minutes. Nurses assessed the patient’s craving, self-efficacy for quitting smoking, motivation for quitting smoking, and depression, and gave advice on how to cope with withdrawal etc. in their behavioral counseling.

### Participants

**Figure 1:**
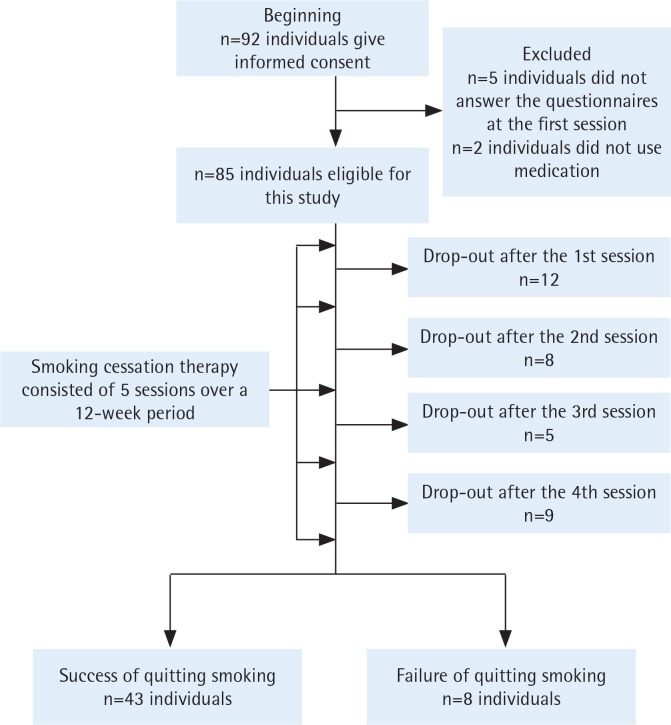
Flow of study subjects

Study subjects were recruited among patients who received SCT for the first time between June 2017 and September 2018. Patients were excluded from the present study if they were not prepared to stop smoking or if they did not have nicotine dependence [i.e. if they had a Tobacco Dependence Screener (TDS)^15^ score <5, or Brinkman Index <200]. Ninety-two patients gave written informed consent to receive SCT in this hospital between May 2017 and December 2018 (Figure 1). Seven patients were excluded from the study for the following reasons: five patients did not answer the questionnaires at the first session of SCT and two patients did not receive the standard medication for smoking cessation. The remaining eighty-five patients were eligible for analysis (Figure 1). Written informed consent was obtained from the patients. This study was approved by the Institutional Review Board of Nagoya Medical Center.

### Measures

#### Sociodemographic and smoking-related data

Sociodemographic data included: age, sex, cohabitation, occupation, and having a present illness. Smoking-related variables included: the Fagerström Test for Nicotine Dependence (FTND), smoking history, and Brinkman Index, which were obtained from self-report questionnaires that were filled in at the first session of the SCT. In the FTND, scores ranged from 0 to 10, and we defined a score of ≥7 as indicating severe nicotine dependence^16^. These variables were chosen based on the Japanese standard manual of smoking cessation treatment^17^.

#### The Center for Epidemiologic Studies Depression Scale (CES-D)

We administered the Center for Epidemiologic Studies Depression Scale (CES-D) before each of the 5 sessions of the SCT to assess the patient’s depression status^18^. In the CES-D, scores ranged from 0 to 60, and patients with scores ≥16 were defined as having depression.

#### Definition of success of quitting smoking

Success of quitting smoking was defined as the condition that subjects self-reported quitting smoking for at least the previous 2 weeks at the last session of the SCT, which was verified by the concentration of exhaled carbon monoxide (CO), as measured by the breath CO monitor (≤7 ppm) (Micro Medical, Hoechberg, Germany).

#### The level of nicotine craving

First, we preliminarily created a new Tobacco Craving Index (TCI) that consisted of two axes based on the experience of two smoking cessation specialists, and administered the TCI at two smoking cessation clinics at hospitals. The combination of the strength of craving and frequency of craving was completed from the complaints and symptoms of patients at the above two hospitals during five sessions of the SCT. After creating the TCI, we provided the TCI to six smoking cessation clinics in Japan and obtained the TCI scores from 991 participants between October 2008 and June 2011^19,20^. After data collection, we made a slight revision in the Japanese expression for the strength of the urge to smoke in the TCI questionnaire.

1) Tobacco Craving Index (TCI)

The TCI consists of two axes: one is strength of craving on a 4-point scale (0: I feel no craving for smoking anymore, 1: I feel a need to put something in my mouth to cope with the craving, 2: I need endurance to cope with the craving. 3: I can hardly continue to stop smoking because of a strong craving); and the other is frequency of craving that a patient feels per day on a 4-point scale (0: 0; 1: <1; 2: 1–3; 3: ≥4 times per day). We developed a craving grade consisting of 4 grades (0–3) by combining the strength and frequency of craving (Appendix, Supplementary file). The questionnaire for the TCI asks patients to assess their craving over the past week.

2) Ten-item version of the Questionnaire on Smoking Urges (QSU-brief)

The QSU-brief 12 is used in clinical settings and had been shortened from the 32-item QSU10. The QSU-brief has been used in numerous studies and it was demonstrated to have excellent reliability (Cronbach’s alpha=0.97)^21,22^. The QSU-brief consists of 10 items and respondents answer each item on a 7-point scale from strongly disagree to strongly agree with higher scores indicating stronger urges. The QSU-brief consists of two factors^23^: one factor is craving associated with positive reinforcement of smoking and the other factor reflects anticipation of negative reinforcement of smoking.

### Variables and statistical analysis

We calculated the Pearson coefficient of correlation between the TCI grade and the QSU-brief score at each of the 5 sessions of SCT. We then calculated mean scores of TCI and QSU-brief from the first session to the last session in patients who succeeded in quitting smoking by the last session of the SCT (Success group) and in patients who did not succeed in quitting smoking by the last session (Failure group). In addition, we calculated the average area under the receiver operating characteristic curve (AUC) between smoking status at the 5th session and both QSU-brief and TCI at each session. At each of the 5 sessions, to clarify how much the TCI grade predicts smoking status at the last session, we performed multivariate logistic regression analysis with adjustment for FTND (≥7 or < 7), CES-D score (≥16 or <16) and each of the two craving indexes separately.

## RESULTS

Table 1 shows the characteristics of the subjects by the grade of TCI at the first session of the SCT. As the TCI grade increased, the mean age of participants decreased. In the presence of a cohabiter, the proportion of those who had a TCI grade of 0 or 1 was 18.4% and the proportion of those with grade 3 was 49.0%. Among the participants having a present illness, the proportion who had grade 0 or 1 of TCI was 20.5% and the proportion who had grade 3 was 42.3%. Higher FTND score and CES-D score were also related to higher grade of craving as assessed by the TCI. Participants who had grade 3 of TCI had a higher mean QSU-brief score than those who had grade 0 or 1 or grade 2.

**Table 1:** Characteristics of the study subjects

*Characteristics*	*Tobacco Craving Index (TCI)*			

	*Grade 0 or 1 n (%)*	*Grade 2 n (%)*	*Grade 3 n (%)*	*Total*
**Sex**				
Female	7 (29.7)	6 (25)	11 (45.8)	24
Male	11 (18.0)	25 (41.0)	25 (41.0)	61
**Age** (years)				
Mean (SD)	63.3 (11.9)	58.2 (14.0)	57.4 (15.8)	58.9 (14.4)
**Experience of quitting smoking**				
Absence	4 (22.2)	6 (33.3)	8 (44.4)	18
Presence	14 (20.9)	25 (37.3)	28 (41.8)	67
**Cohabitation**				
Absence	9 (25.0)	15 (41.7)	12 (33.3)	36
Presence	9 (18.4)	16 (32.7)	24 (49.0)	49
**Occupation**				
Absence	13 (24.5)	15 (28.3)	25 (47.2)	53
Presence	5 (15.6)	16 (50.0)	11 (34.4)	32
**Having a present illness**				
Absence	2 (28.6)	2 (28.6)	3 (42.8)	7
Presence	16 (20.5)	29 (37.2)	33 (42.3)	78
**FTND**				
<7	14 (25.5)	21 (38.1)	20 (36.4)	55
t7	4 (13.3)	10 (33.3)	16 (53.3)	30
**CES-D at the first session**				
<16	13 (27.7)	19 (40.4)	15 (31.9)	47
t16	5 (13.2)	12 (31.6)	21 (55.2)	38
QSU-brief *				
Mean (SD)	31.1 (16.0)	31.0 (14.8)	41.8 (16.0)	35.6 (16.3)

* The scores on the 10-item Questionnaire on Smoking Urges (QSU-brief) at the first session are shown.

FTND: Fagerström Test for Nicotine Dependence. CES-D: Center for Epidemiological Studies Depression scale.

The correlation coefficient between the TCI grade and QSU-brief score in the first to the fifth session of SCT was 0.27, 0.55, 0.72, 0.58 and 0.68, respectively (Table 2). Although they were all statistically significant, low correlation was observed at the first session (0.27). After the first session, the TCI grade had a strong correlation with the QSU-brief score (Table 2).

**Table 2:** Correlation between the TCI grade and QSU-brief score at each of the five sessions in the Japanese SCT

	*TCI*	*1st (n=85)*	*2nd (n=73)*	*3rd (n=65)*	*4th (n=60)*	*5th (n=51)*
**QSU-brief**						
**1st**		0.27**				
**2nd**			0.55**			
**3rd**				0.72**		
**4th**					0.58**	
**5th**						0.68**

Pearson’s correlation coefficients are shown.

** p<0.001.

**Figure 2:**
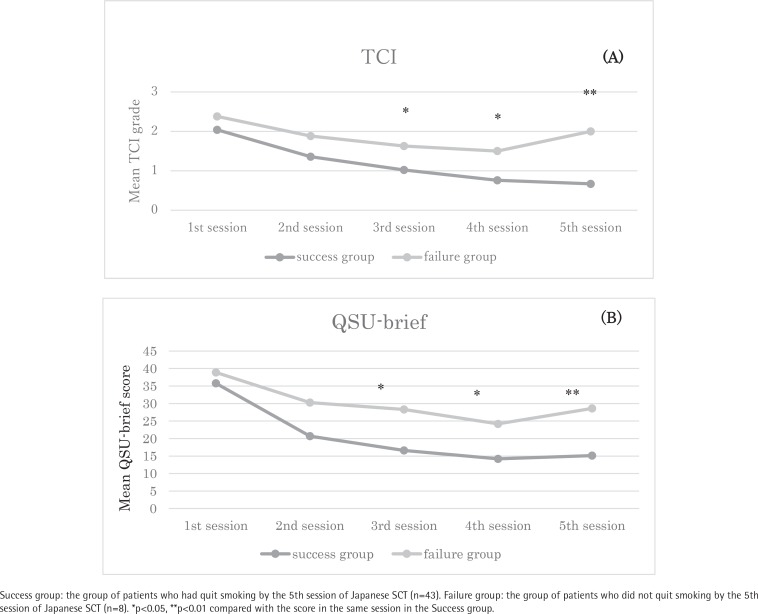
Changes in the (A) mean TCI grade and (B) mean QSU-brief score during the SCT in patients who did or did not quit smoking by the 5th session of the SCT Success group: the group of patients who had quit smoking by the 5th session of Japanese SCT (n=43). Failure group: the group of patients who did not quit smoking by the 5th session of Japanese SCT (n=8). *p<0.05, **p<0.01 compared with the score in the same session in the Success group.

Figures 2A and 2B show the mean TCI grades (Figure 2A) and QSU-brief scores (Figure 2B) from the first to last sessions of the SCT in the two groups of patients who succeeded or failed in quitting smoking at the last session of the SCT. Forty-three patients (Success group) succeeded in quitting smoking at the 5th session, while 8 patients (Failure group) failed to quit smoking by the 5th session. The TCI grade in the 1st session was not significantly different between the Success and Failure groups (Figure 2A). The mean TCI grade in the Success group decreased over the 5 sessions (2.04, 1.36, 1.02, 0.76 and 0.67 in the first to fifth session, respectively), whereas the mean TCI grade in the Failure group did not show a clear declining trend. Finally, the TCI grade in the Success group was significantly lower than that in the Failure group at the 3rd, 4th and last sessions. Regarding the QSU-brief scores, similar tendencies were observed in the change in mean QSU-brief scores with statistically significant differences at the 3rd, 4th and last sessions (Figure 2B) (mean QSU brief score: 35.8, 20.7, 16.6, 14.2 and 15.1 at the first to fifth sessions in the Success group; 38.9, 30.3, 28.3, 24.2 and 28.6 in the Failure group, respectively).

The AUC values between the TCI grade at each session and success of quitting smoking were 0.615, 0.676, 0.667, 0.702 and 0.881 in the first to fifth sessions, respectively. On the other hand, the AUC values between the QSU-brief score at each session and the success of quitting smoking were 0.536, 0.699, 0.709, 0.737 and 0.88, respectively (Table 3).

**Table 3:** Average area under the receiver operating characteristic curve (AUC) of the TCI grade or QSU-brief score in predicting the success of quitting smoking at each of the five sessions in the Japanese SCT

*Session*	*TCI*		*QSU-brief*	

	*AUC*	*95% CI*	*AUC*	*95% CI*
**1st**	0.615	(0.410–0.820)	0.536	(0.343–0.729)
**2nd**	0.676	(0.500–0.851)	0.699	(0.451–0.947)
**3rd**	0.667	(0.443–0.891)	0.709	(0.487–0.932)
**4th**	0.702	(0.508–0.897)	0.737	(0.540–0.933)
**5th**	0.881	(0.777–0.985)	0.849	(0.739–0.958)

Table 4 shows the odds ratio for success of quitting smoking for the sequential scores of TCI and QSU-brief at each session. Both the TCI grade and QSU-brief score after the second session significantly reflected the success of quitting smoking. Participants who had higher TCI grade or QSU-brief score showed lower probability of success of quitting smoking.

**Table 4:** Adjusted odds ratio, and 95% CI, for success of quitting smoking at the last session of the SCT for TCI grade and QSU-brief score assessed from the 1st to the last session in the Japanese SCT (n=51)

	*1st*	*2nd*	*3rd*	*4th*	*5th*
**TCI grade**	0.51 (0.16–1.62)	0.25 (0.06–1.11)	0.33 (0.11–0.93)*	0.22 (0.06–0.77)*	0.24 (0.07–0.82)**
**QSU–brief score**	0.97 (0.92–1.04)	0.94 (0.89–1.00)	0.92 (0.86–0.99)*	0.92 (0.86–0.99)*	0.93 (0.87–0.99)*

Adjusted for FTND score and CES–D score at the first session of SCT.

* p<0.05.

** p<0.01.

## DISCUSSION

We previously created a new indicator to assess the degree of craving for smoking in patients who underwent the SCT, which we called TCI, and found that the TCI grade showed a significant inverse association with the probability of success of quitting smoking in Japanese SCT patients. The strength of the association between the TCI grade and probability of success of quitting smoking was similar to that between the QSU-brief score and probability of success of quitting smoking. Our results showed that the TCI grade and QUS-brief score were positively correlated with each other in each of the 5 sessions of the SCT. In addition, the AUC scores between the TCI grade or QSU-brief score and success of quitting smoking were similar in each session of the SCT. Cox et al.^9^ administered the QSU-brief to 221 active smokers in the laboratory setting and to 112 smokers enrolled in a comprehensive smoking cessation program. They demonstrated that the QSU-brief was predictive of the general level of craving in active smokers both in the laboratory and in the clinical setting^12^. The positive correlation between the TCI grade and QSU-brief score indicates that the TCI is a useful indicator. In addition, the TCI questionnaire is easy to administer in the clinical setting.

The change in TCI grade from the first to last session of the SCT was similar with the change in QSU-brief score in the Success group and also in the Failure group. In addition, multivariate logistic regression analysis showed that the TCI grade and QSU-brief score assessed after the second session similarly reflected the success of quitting smoking. Schnoll et al.^24^ examined longitudinal changes in craving using the QSU-brief from the baseline assessment to the 6-month outcome following 8 weeks of smoking cessation treatment and counseling. The results demonstrated that the craving scores of both smokers and abstainers declined after 8 weeks from baseline, and thereafter the mean craving score of abstainers became sharply lower compared with that of smokers. Finally, those who could not stop smoking showed higher QSU-brief scores compared with the abstainers^24^, similar to the results in our study. They mentioned that reduction in the QSU-brief score during the first 8 weeks was significantly associated with lower risk of subsequent relapse^24^. Our study had a shorter observation period than the study of Schnoll et al.^24^. However, the changes in TCI grade and QUS-brief score through the SCT in the Success and Failure groups in our study showed similar trends as those in the Schnoll et al.^24^ study and suggested that a reduction in craving score is associated with success of quitting smoking in smoking cessation intervention. These results suggest that the TCI is a useful index for predicting smoking status similar to the QSU-brief.

### Strengths and limitations

One strength of this study was that we evaluated the strength of the association between the TCI grade and smoking behavior and compared it with the association between the score on the QSU-brief that is widely used throughout the world for smoking behavior. On the other hand, this study has potential limitations. First, this study examined patients visiting one smoking cessation clinic at a general hospital. Many of this clinic’s patients have a physical comorbidity unlike the general population. This difference possibly attenuated the representativeness of the general population. Therefore, further studies to assess the predictability among smokers without comorbidities are needed. Second, our study had a small number of subjects: among the 85 patients who attended the first session of the SCT, 34 dropped out and 51 remained at the end of the study. This might have resulted in lower statistical power to assess the relationship between craving score and smoking status; the craving scores at the first and second sessions did not reflect smoking cessation statistically in our study. However, our study demonstrated that the mean TCI grade changed in a similar manner as the mean QSU-brief score through the 5 sessions of the SCT and predicted smoking status at the last session as in previous studies^10,12^. Our findings indicate that the TCI is a useful indicator to assess tobacco craving in patients undergoing SCT. The TCI grade may be used to adjust the volume and duration of medication for smoking cessation treatment, or to adjust the duration of cognitive behavioral therapy in nurses’ counseling.

## CONCLUSIONS

Our study showed that the new craving indicator called TCI, as well as QSU-brief, can be used as a predictive tool for success of quitting smoking in the Japanese SCT. As the TCI consists of only two questionnaire items, it may be easily used in smoking cessation intervention settings. Further studies are needed to validate this finding in other populations.
