# Supplementary material for: Development of a new craving index for anticipating quitting smoking in patients who undergo the Japanese smoking cessation therapy
Source: Tob Induc Dis. 2019 Dec 3;17:89. doi: 10.18332/tid/114164 (PMC6903806; doi:10.18332/tid/114164)
Supplement: Supplementary file 1 [file TID-17-89-s1.pdf]

Supplementary file, Document 1

Ten item version of the Questionnaire on Smoking Urges (QSU-brief)

(1=strongly disagree to 7=strongly agree)

1. I have a desire for a cigarette right now.
2. Nothing would be better than smoking a cigarette right now.
3. If it were possible, I would probably smoke now.
4. I could control things better right now if I could smoke.
5. All I want right now is a cigarette.
6. I have an urge for a cigarette.
7. A cigarette would taste good now.
8. I would do almost anything for a cigarette now.
9. Smoking would make me less depressed.
10. I am going to smoke as soon as possible.

Tobacco Craving Index (TCI)

Level of craving	0: I have no craving for smoking at all<br>1: I miss having something in my mouth<br>2: I can manage the craving<br>3: I keep having craving for smoking
Frequency of daily craving	0: not at all　1: less than once a day<br>2: 1~3 times a day　3: 4 times or more a day

The patient was asked to rate their level of craving on a scale of 0-3 and frequency of daily craving on a scale of 0-3. The TCI grade (G) was determined by the patient's ratings on the two questionnaire items according to the following chart. The TCI grade (G) ranges from 0 to 3.

TCI Grade

Level of craving (columns) / **Frequency of daily craving** (rows)

	**0**	**1**	**2**	**3**
**0**	**G0**			
**1**		**G1**	**G1**	**G2**
**2**		**G1**	**G2**	**G3**
**3**		**G1**	**G2**	**G3**

© 2019 Taniguchi C. et al.
